# Metformin for Preventing Progression From Prediabetes to Diabetes Mellitus in People Living With Human Immunodeficiency Virus

**DOI:** 10.7759/cureus.24540

**Published:** 2022-04-27

**Authors:** Hataikarn Nimitphong, Sitta Jiriyasin, Pisekporn Kasemasawachanon, Somnuek Sungkanuparph

**Affiliations:** 1 Department of Medicine, Faculty of Medicine Ramathibodi Hospital, Mahidol University, Bangkok, THA; 2 Chakri Naruebodindra Medical Institute, Faculty of Medicine Ramathibodi Hospital, Mahidol University, Samut Prakan, THA

**Keywords:** randomized control trial, insulin resistance, metformin, diabetes, prediabetes, hiv

## Abstract

Background

Diabetes mellitus (DM) and human immunodeficiency virus (HIV) itself increase the risk for cardiovascular diseases in people living with HIV (PLHIV). Prediabetes, a condition preceding DM, is common in PLHIV receiving antiretroviral therapy (ART). Both metformin and lifestyle interventions have been established to reduce the risk of progression from prediabetes to DM in the general population. This study aimed to evaluate the efficacy of metformin for preventing DM in prediabetic PLHIV.

Methods

An open-label randomized controlled clinical trial was conducted in HIV-positive persons with prediabetes. The participants were randomized into two groups: the metformin group (received metformin) and the control group (did not receive metformin). All participants were counseled regarding diet control and lifestyle modification and followed for 12 months. The primary endpoint was the development of DM. Fasting plasma glucose (FPG), two-hour plasma glucose (2-h PG) after 75 g oral glucose tolerance test (OGTT), hemoglobin A1c (HbA1c), and computer-based homeostatic model assessment index of beta-cell function (HOMA%B) and insulin resistance (HOMA-IR) were analyzed.

Results

Seventy-four participants were enrolled, 37 in each group. The mean age was 49.6 years, and 68.9% were males. At baseline, the mean CD4 cell count was 570 cells/mm^3^, and the mean body mass index (BMI) was 24.6 kg/m^2^. Baseline characteristics including age, sex, BMI, waist/hip ratio, duration of ART, ART regimen, CD4 cell count, and HIV RNA were similar between the two groups. The mean FPG, 2-h PG, HbA1c, HOMA%B, and HOMA-IR at baseline were also similar between the two groups. At 12 months, one participant in the metformin group and three in the control group developed DM (risk reduction: 5.41%; 95% confidence interval (CI): −6.92%-18.78%). When we compared changes in parameters between the two groups, there were trends toward more changes in HbA1c ($\Delta$HbA1c) at both six months (metformin group versus control group: -0.17% ± 0.20% versus 0.02% ± 0.58%; p = 0.074) and 12 months (metformin group versus control group: -0.05% ± 0.23% versus 0.06% ± 0.27%; p = 0.065). When we considered changes in all parameters in each group, the metformin group had significant reductions in body weight (BW) and BMI at both six and 12 months, and significant reductions in HbA1c and HOMA-IR at six months. No participant had adverse effects that led to the discontinuation of metformin. No cardiovascular event was observed during the study period.

Conclusions

Metformin tends to improve HbA1c and insulin resistance and may prevent progression from prediabetes to DM in HIV-positive persons with prediabetes. A further large study with a longer study period is needed to evaluate the long-term benefit of metformin.

## Introduction

At the end of 2020, an estimated 38 million people globally were living with the human immunodeficiency virus (HIV). This magnitude of HIV infection continues to be a major health problem worldwide. Although antiretroviral therapy (ART) dramatically reduces morbidity and mortality from opportunistic diseases in people living with HIV (PLHIV), cardiovascular disease and other metabolic diseases are common causes of non-AIDS-related mortality [1,2].

Type 2 diabetes mellitus (T2DM) is a leading health problem worldwide and is a major risk factor for cardiovascular diseases [3]. People living with HIV (PLHIV) are more likely to have T2DM than the general population [4-7]. Additionally, some HIV medicines may increase the risk of T2DM in PLHIV. The risk of cardiovascular diseases in PLHIV is, therefore, greater than that in the general population.

Prediabetes is a health condition where plasma glucose (PG) levels are higher than normal, but not high enough yet to be diagnosed as T2DM. Prediabetes leads to an increased risk of developing T2DM and cardiovascular diseases [8]. Approximately 5%-10% of people with prediabetes progress to T2DM annually [9,10]. Accordingly, preventing the progression of prediabetes to T2DM is expected to lower the risk of cardiovascular diseases in this population.

With regard to the Diabetes Prevention Program (DPP), intensive lifestyle intervention is the most effective strategy to reduce the risk of developing T2DM in the general population [11]. The administration of metformin in people with prediabetes can also reduce the risk, however, with less effectiveness. To date, strategies to delay or prevent prediabetes from progressing into T2DM in PLHIV are still lacking. We hypothesize that in PLHIV with prediabetes, metformin would be able to reduce the risk of developing T2DM and subsequently reduce risk factors of cardiovascular diseases and other diabetes-related morbidities.

This study aimed to compare the occurrence of T2DM in PLHIV with prediabetes who receive metformin therapy with those in a control group. The secondary objectives were to compare parameters of glucose metabolism and insulin resistance.

The preliminary results of this article were previously presented as a poster exhibition (abstract number 2250) at the 2018 ID Week, an annual scientific meeting of the Infectious Diseases Society of America, on October 2-7, 2018.

## Materials and methods

Study design

This study was an open-label prospective randomized trial. PLHIV who visited an infectious disease clinic in a medical school hospital were recruited and followed up for 12 months. The inclusion criteria were HIV-positive persons aged 35-60 years old and who met all of the four following conditions: (1) prediabetes (according to the criteria of the American Diabetes Association (ADA), defined by impaired fasting glucose (IFG) (fasting plasma glucose (FPG): 100 mg/dL (5.6 mmol/L)-125 mg/dL (6.9 mmol/L)), impaired glucose tolerance (IGT) (two-hour plasma glucose (2-h PG) after a 75 g oral glucose tolerance test (OGTT): 140 mg/dL (7.8 mmol/L)-199 mg/dL (11.0 mmol/L)), or glycosylated hemoglobin (HbA1c) of 5.7%-6.4%) [12]; (2) presence of at least one of the following conditions for the previous prediabetes treatment suggestion by the ADA: hypertension (sitting blood pressure > 135/85 mmHg), low HDL cholesterol (<40 mg/dL in females and <35 mg/dL in males), elevated TG (≥150 mg/dL), a family history of diabetes in a first-degree relative, or a history of gestational diabetes mellitus (GDM) [13]; and (3) able to sign an informed consent. The exclusion criteria were prior diagnosis with cardiovascular disease, T2DM, pregnant, receiving drugs that altered glucose tolerance, having contraindications for metformin use, an estimated glomerular filtration rate below 45 mL/minute/1.73 m^2^, or refusing informed consent.

The study was reviewed and ethically approved by the Committee on Human Rights Related to Research Involving Human Subjects, Faculty of Medicine Ramathibodi Hospital, Mahidol University. The approval number was MURA2016/573. All participants provided written informed consent.

Randomization and intervention

The participants were randomly assigned to receive either metformin at a dose of 500 mg taken orally twice a day (metformin group) or none (control group). Adherence to the treatment regimen was assessed by pill count. The standard lifestyle recommendations including weight reduction and increased physical activity were provided to all participants. After randomization into two groups of treatment, the participants were asked to return to the clinic every three months and were followed for 12 months.

Recorded data included demographic characteristics, duration of known HIV infection, AIDS-defining illness, duration of ART, antiretroviral regimens, and adverse events. Waist circumference (WC), hip circumference (HC), body weight (BW), body mass index (BMI), and blood pressure were assessed during every visit.

Biochemical measurement

All participants arrived at the clinic in the morning after at least an eight-hour overnight fast. The 75 g OGTT was performed at baseline, and six and 12 months. Glucose levels were measured at fasting and two-hour post-oral glucose loading. Fasting blood samples were also measured for HbA1c and insulin. Plasma glucose and HbA1c were measured using a Dimension® RxL Max® analyzer (Siemens Healthcare Diagnostics, Tarrytown, NY, USA). Plasma insulin was measured using the immunochemiluminescence method (Siemens Healthcare Diagnostics, Tarrytown, NY, USA) using an automated machine. The computer-based homeostatic model assessment index of beta-cell function (HOMA%B) and computer-based homeostatic model assessment index of insulin resistance (HOMA-IR) were calculated using a homeostasis model assessment-2 (HOMA-2) calculator (www.dtu.ox.ac.uk/homa) [14]. The disposition index was calculated as HOMA%B divided by HOMA-IR. According to the HOMA-2 calculator, the accepted insulin levels are 20-400 pmol/L. We found that 21 participants had insulin levels < 20 pmol/L. Therefore, HOMA%B and HOMA-IR were calculated in 53 participants (24 participants in the metformin group and 29 participants in the control group).

Outcomes

The primary outcome was the occurrence of T2DM in HIV-positive individuals with prediabetes at six and 12 months diagnosed using the 2017 criteria of the American Diabetes Association (a value for plasma glucose ≥ 126 mg/dL or 2-h PG ≥ 200 mg/dL or HbA1c ≥ 6.5% or a random plasma glucose ≥ 200 mg/dL with the presence of classic symptoms of hyperglycemia). The diagnosis required confirmation by a second test, either the same or a different test, except in participants who presented with classic symptoms [12]. The secondary outcomes were FPG, 2-h PG, HOMA%B, HOMA-IR, and disposition index at six and 12 months, and the occurrence of cardiovascular disease and microvascular complications of T2DM at 12 months.

Statistical analysis

The sample size was calculated using a simple formula for pilot studies [15]. We used the problem probability based on the cumulative incidence of diabetes mellitus at one year from a previous study [11]. A number of 74 participants, 37 in each treatment group, was needed. We, therefore, enrolled 74 participants in this study and randomly assigned (1:1) them into two groups, one for metformin therapy, and the other as a control group, by computer-generated random numbers. In the analysis to compare the variables between two groups, the Student t-test or Mann-Whitney U test was used for continuous variables; a chi-square or Fisher’s exact test was used for categorical variables, where appropriate. The probability of occurrence of diabetes mellitus was estimated using Kaplan-Meier analysis and compared between groups using the log-rank test. Absolute risk reduction of diabetes mellitus occurrence with 95% confidence interval (CI) was used to determine the effect of metformin therapy. A comparison of metabolic parameters in each group at baseline and six months, and baseline and 12 months was performed using a paired-samples t-test. A comparison of changes in metabolic parameters over time between the two groups was performed using the Student t-test. Analyses were performed using SPSS statistical software package version 18.0 (SPSS Inc., Chicago IL, USA).

## Results

Baseline characteristics

Seventy-four participants were studied. There were 37 participants in each study group. Fifty-one (68.9%) participants were male, and the mean age was 49.6 ± 6.4 years. The mean BMI was 24.6 ± 3.6 kg/m^2^. The mean duration of known HIV infection was 14.7 ± 18.2 years. All participants were on ART with undetectable HIV RNA, and the mean CD4 cell count was 569.6 ± 239.0 cells/mm^3^. Of all, 86 (81.1%) participants had received non-nucleoside reverse transcriptase inhibitor (NNRTI)-based antiretroviral regimens; the rest were on protease inhibitor (PI)-based regimens. All baseline demographic data and characteristics including age, sex, duration of known HIV infection, CD4 cell counts, duration and types of ART regimens, BW, BMI, WC, waist/hip ratio, and lipid profiles were similar between the two groups (Table 1). With regard to glucose homeostasis, FPG, 2-h PG, HbA1c, HOMA%B, HOMA-IR, and disposition index were all similar between the two groups.

**Table 1:** Baseline characteristics between participants in metformin and control groups ART: antiretroviral therapy; NNRTI: non-nucleoside reverse transcriptase inhibitor; PI: protease inhibitor; BW: body weight; BMI: body mass index; FPG: fasting plasma glucose; 2-h PG: two-hour plasma glucose; HbA1c: hemoglobin A1c; HOMA%B: homeostatic model assessment index of beta-cell function; HOMA-IR: homeostatic model assessment index of insulin resistance ^a^n = 24; ^b^n = 29

Characteristics	Metformin group (N = 37)	Control group (N = 37)
Gender, number (%)		
Male	26 (70.3)	25 (67.6)
Female	11 (29.7)	12 (32.4)
Age, years, mean ± SD	50.7 ± 6.9	48.4 ± 5.8
Duration of HIV infection, years, mean ± SD	14.3 ± 18.4	15.1 ± 18.3
AIDS-defining illness, number (%)	15 (40.5)	11 (30.6)
Duration of ART, years, mean ± SD	10.9 ± 5.6	12.7 ± 6.7
Types of ART regimens, number (%)		
NNRTI-based regimen	31 (83.8)	29 (78.4)
PI-based regimen	6 (16.2)	8 (21.6)
CD4 cell counts, cells/mm^3^, mean ± SD	537.5 ± 240.8	601.8 ± 236.0
HIV RNA < 40 copies/mL, number (%)	37 (100)	37 (100)
BW, kg, mean ± SD	67.8 ± 13.6	66.5 ± 13.0
BMI, kg/m^2^, mean ± SD	24.9 ± 3.7	24.2 ± 3.6
WC, cm, mean ± SD	88.7 ± 10.0	86.4 ± 10.3
Waist/hip ratio, mean ± SD	0.908 ± 0.05	0.902 ± 0.07
Family history of diabetes, number (%)	34 (91.9)	30 (81.1)
Hypertension, number (%)	14 (37.8)	12 (32.4)
Low HDL cholesterol, number (%)	5 (13.5)	8 (21.6)
Elevated triglycerides, number (%)	4 (10.8)	8 (21.6)
Aspartate transaminase, U/L, mean ± SD	31.3 ± 10.2	32.1 ± 12.6
Alanine transaminase, U/L, mean ± SD	38.2 ± 16.2	39.4 ± 18.1
FPG, mg/dL, mean ± SD	95.32 ± 9.70	95.95 ±7.67
2-hr PG, mg/dL, mean ± SD	123.70 ± 42.63	121.78 ± 32.24
HbA1c, %, mean ± SD	5.68 ± 0.34	5.61 ± 0.38
HOMA%B, mean ± SD	112.43 ± 41.60^a^	117.59 ± 64.40^b^
HOMA-IR, mean ± SD	1.49 ± 0.75^a^	1.75 ± 1.28^b^
Disposition index, mean ± SD	81.61 ± 19.61^a^	78.72 ± 23.26^b^

The occurrence of diabetes mellitus

At six months, none in the metformin group and one participant in the control group developed DM (by HbA1c criteria) (risk reduction: 2.70%; 95% confidence interval: −6.96%-13.82%). At 12 months, one participant (by FPG and 2-h PG criteria) in the metformin group and three in the control group (by all FPG, 2-h PG, and HbA1c criteria) developed DM (risk reduction: 5.41%; 95% CI: −6.92%-18.78%).

Anthropometric measurements

At six months, when compared to baseline, significant reductions in BW (0 versus six months: 67.8 ± 13.6 versus 66.6 ± 13.4 kg; p = 0.019) and BMI (0 versus six months: 24.9 ± 3.6 versus 24.5 ± 3.7 kg/m^2^; p = 0.024) were detected in the metformin group, whereas there were no changes of those parameters in the control group. However, the changes in BW ($\Delta$BW) and BMI ($\Delta$BMI) of the metformin group were not significantly different when compared with the control group (Table 2). There were no changes in WC in both groups.

At 12 months, in the metformin group, BW (0 versus 12 months: 67.8 ± 13.6 versus 66.5 ± 13.7 kg; p = 0.004) and BMI (0 versus 12 months: 24.9 ± 3.6 versus 24.4 ± 3.6 kg/m^2^; p = 0.002) significantly decreased when compared to baseline, while there was no change in WC. In the control group, BW, BMI, and WC did not change over time. Similar to the results at six months, $\Delta$BW, $\Delta$BMI, and the changes in WC ($\Delta$WC) between the two groups were comparable (Table 2).

**Table 2:** Mean changes of glycemic profiles, beta-cell function, and insulin resistance compared between metformin and control groups $\Delta$: changes; BW: body weight; BMI: body mass index; WC: waist circumference; FPG: fasting plasma glucose; 2-h PG: two-hour plasma glucose; HbA1c: hemoglobin A1c; HOMA%B: homeostatic model assessment index of beta-cell function; HOMA-IR: homeostatic model assessment index of insulin resistance

Outcomes (mean ± SD)	Metformin group (N = 37)	Control group (N = 37)	P-value
Anthropometric measurements			
At six months			
$\Delta$BW, kg	-1.18 ± 2.93	-0.19 ± 2.20	0.103
$\Delta$BMI, kg/m^2^	-0.43 ± 1.10	-0.07 ± 0.82	0.122
$\Delta$WC, cm	0.96 ± 4.96	2.05 ± 3.77	0.292
At 12 months			
$\Delta$BW, kg	-1.34 ± 2.63	-0.55 ± 2.60	0.193
$\Delta$BMI, kg/m^2^	-0.52 ± 0.95	-0.20 ± 0.97	0.157
$\Delta$WC, cm	0.87 ± 4.69	1.86 ± 4.25	0.345
Glycemic changes			
At 6 months			
$\Delta$FPG, mg/dL	4.16 ± 7.91	3.46 ± 6.82	0.684
$\Delta$2-h PG, mg/dL	15.30 ± 42.21	14.76 ± 40.80	0.955
$\Delta$HbA1c, %	-0.17 ± 0.20	0.02 ± 0.58	0.074
At 12 months			
$\Delta$FPG, mg/dL	7.70 ± 10.24	6.76 ± 17.53	0.778
$\Delta$2-h PG, mg/dL	22.08 ± 41.98	22.73 ± 58.64	0.957
$\Delta$HbA1c, %	-0.05 ± 0.23	0.06 ± 0.27	0.065
	Metformin group (N = 24)	Control group (N = 29)	P-value
Glucose homeostasis			
At six months			
$\Delta$HOMA%B	-26.01 ± 33.57	-11.84 ± 41.98	0.187
$\Delta$HOMA-IR	-0.32 ± 0.61	-0.05 ± 0.95	0.224
$\Delta$Disposition index	0.71 ± 22.46	-7.99 ± 22.49	0.167
At 12 months			
$\Delta$HOMA%B	-26.13 ± 34.80	-13.53 ± 61.63	0.378
$\Delta$HOMA-IR	-0.20 ± 0.66	0.05 ± 1.43	0.441
$\Delta$Disposition index	-9.29 ± 20.68	-0.21 ± 52.03	0.426

Glycemic changes and glucose homeostasis

**Figure 1:**
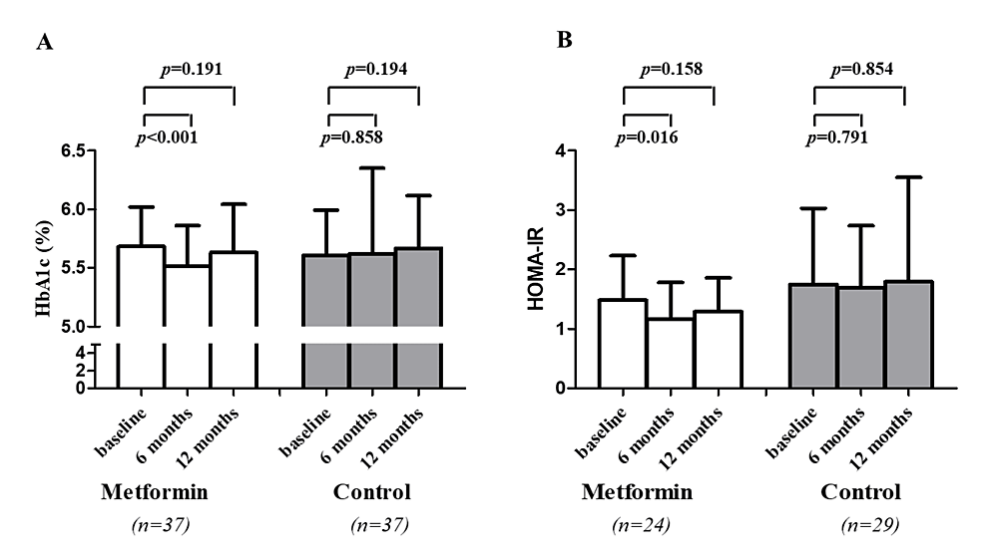
Comparison of HbA1c and HOMA-IR at baseline, and six and 12 months stratified by the treatment group HbA1c: hemoglobin A1c; HOMA-IR: homeostatic model assessment index of insulin resistance

At six months, significant increases in FPG (0 versus six months: 95.32 ± 9.70 versus 99.49 ± 9.43 mg/dL, p = 0.003, in the metformin group, and 95.95 ± 7.67 versus 99.41 ± 7.99 mg/dL, p = 0.004, in the control group) and 2-h PG (0 versus six months: 123.70 ± 42.63 versus 139.00 ± 46.92 mg/dL, p = 0.034, in the metformin group, and 121.78 ± 32.24 versus 136.54 ± 49.29 mg/dL, p = 0.034, in the control group) were demonstrated in both groups. In the metformin group, HbA1c significantly decreased from baseline (0 versus six months: 5.68% ± 0.34% versus 5.52% ± 0.34%; p < 0.001) (Figure 1A). In the control group, HbA1c did not change at six months (Figure 1A). Furthermore, we compared the changes in those parameters among the two groups. Changes in FPG ($\Delta$FPG) and 2-h PG ($\Delta$2-h PG) were similar between the two groups. However, changes in HbA1c ($\Delta$HbA1c) had a trend toward more changes in the metformin group (p = 0.074) (Table 2).

Similarly, at 12 months, significant increases in FPG (0 versus 12 months: 95.32 ± 9.70 versus 103.03 ± 12.99 mg/dL, p < 0.001, in the metformin group, and 95.95 ± 7.67 versus 102.70 ± 18.26 mg/dL, p = 0.025, in the control group) and 2-h PG (0 versus 12 months: 123.70 ± 42.62 versus 145.78 ± 55.33 mg/dL, p = 0.003, in the metformin group, and 121.78 ± 32.24 versus 144.51 ± 69.94 mg/dL, p = 0.024, in the control group) were found in both groups. $\Delta$FPG and $\Delta$2-h PG were not different between the two groups (Table 2). Overtime, a nonsignificant reduction in HbA1c was detected in the metformin group, while a nonsignificant increase in HbA1c was detected in the control group (Figure 1A). Accordingly, $\Delta$HbA1c had a trend toward more change in the metformin group (p* *= 0.065) (Table 2).

With regard to insulin secretion and resistance, there were no differences in $\Delta$HOMA%B, $\Delta$HOMA-IR, and $\Delta$disposition index between the two groups at both six and 12 months (Table 2). When we considered changes in glucose homeostasis over time, at six months, HOMA-IR was significantly decreased in the metformin group, while there was no change in HOMA-IR in the control group (Figure 1B). However, at 12 months, there were no changes in HOMA-IR in both groups (Figure 1B).

The occurrence of cardiovascular disease and the microvascular complications

At 12 months, there were no differences in the occurrence of cardiovascular disease and the occurrence of microvascular complications of T2DM in HIV-infected patients in the metformin and control groups.

## Discussion

Our study shows that in PLHIV with prediabetes, diabetes tended to occur in the control group more frequently than in the metformin group. When compared to the control group, participants who received metformin had a nonsignificantly lower $\Delta$HbA1c at both six and 12 months. Nonetheless, the changes in all anthropometric measurements, FPG, 2-h PG, and all parameters of glucose homeostasis in the metformin and control group were not statistically different. When considered in the individual group of treatment, treatment with metformin could decrease BW and BMI at six months, which were maintained at 12 months. In addition, significant reductions in HbA1c and HOMA-IR were found at six months. There were no reports of the occurrence of cardiovascular disease and microvascular complications in the present study.

Treatment with metformin tended to delay the occurrence of T2DM and reduced BW, BMI, HbA1c, and HOMA-IR in PLHIV, and these effects were similar to those in non-HIV-infected patients [11,16,17]. For example, a recent meta-analysis of randomized trials of metformin for the prevention of diabetes in high-risk individuals showed that metformin decreased new-onset diabetes compared with standard diet and exercise by 50% (risk ratio: 0.5; 95% CI: 0.38-0.65) [17]. The largest trial in the mentioned meta-analysis is the Diabetes Prevention Program (DPP). In this landmark trial, at an average follow-up of three years, metformin and intensive lifestyle intervention demonstrated a reduction in the rate of progression to diabetes compared with placebo by 31% and 58%, respectively [11]. The subsequent 15-year follow-up study reported that the incidences of diabetes development were lower among those in the original metformin group compared with the placebo group by 17% and 36% when the diagnosis was based on an FPG and/or 2-h PG and HbA1c, respectively. However, the beneficial effect of metformin for diabetic prevention is not equal in all subjects. Additional analyses revealed that metformin had a greater selective effect in those who were more obese, had higher fasting glucose, had a history of gestational diabetes, or were younger [11,18-21]. Therefore, lower BMI and lower FPG and HbA1c at baseline in our participants when compared to those in the aforementioned studies could partly explain the less promising results in the present study. The different dosage of the medication (i.e., 1,000 mg versus 1,700 mg per day in ours versus the DPP study, respectively) and other baseline characteristics (general population versus PLHIV) could be other reasons. More importantly, this result may be underpowered due to the small sample size and relatively short duration of follow-up.

Metformin works mainly by reducing gluconeogenesis and opposing glucagon-mediated signaling in the liver and, to a lesser extent, by increasing glucose uptake in the skeletal muscle [22]. In addition, according to diabetes prevention, weight loss was an important determinant. Accordingly, our findings supported the potential benefit of metformin in this particular population. Only in the metformin group, a significant reduction in BW and HOMA-IR was found. These would lead to a significant reduction in HbA1c after receiving the metformin treatment. However, after 12 months, we could only demonstrate a trend of lower $\Delta$HbA1c in the metformin group. For the cardiovascular disease and microvascular complication effects, receiving metformin did not provide those benefits in our cohort. This corresponded with other current evidence in the general population with prediabetes, showing that data on mortality, and macrovascular and microvascular diabetic complications were sparse or missing [17]. Currently, some guidelines in the general population [23] support the therapeutic use of metformin for diabetes prevention in defined circumstances, favoring the use of metformin alongside lifestyle change for younger subjects with higher levels of BMI. A general prescription of metformin in all people, particularly in PLHIV, with prediabetes should not be applied at the current time. A large phase 3 randomized placebo-controlled double-blind trial of metformin in PLHIV with prediabetes (META Phase 3) is now ongoing [24].

The limitation of our study is that our study had a relatively small sample size and short duration. Lifestyle changes of participants may be another important confounder that may lessen the apparent effect of metformin. In a real-world setting, patients may not be very compliant with lifestyle changes, and the benefit of metformin may become more evident. Furthermore, patients were not blinded to the intervention that they received because we did not provide placebo tablets for them. In addition, our study did not have an accurate parameter to evaluate the effects of lifestyle changes in both groups.

## Conclusions

In summary, metformin tends to improve HbA1c and insulin resistance and may prevent progression from prediabetes to DM in HIV-positive persons with prediabetes. Further research with a large-scale population of PLHIV and a longer study time is warranted to evaluate the long-term benefit of metformin in PLHIV with prediabetes.
